# Evaluating the delivery of care by telemedicine for incarcerated people living with HIV: a cohort study

**DOI:** 10.1186/s12879-024-09528-1

**Published:** 2024-07-22

**Authors:** Ruth C. Dunn, Cassidy J. Stegall, Colten Creel, Christian J. Fuchs, Barbara E. Menzies, Nathan A. Summers

**Affiliations:** 1 Department of Medicine, University of Tennessee Health Science Center College of Medicine, Memphis, TN USA; 2 Department of Medicine, Division of Infectious Diseases, University of Tennessee Health Science Center, Memphis, TN USA

**Keywords:** HIV, Hepatitis C, Telehealth, Telemedicine, HIV care continuum

## Abstract

**Background:**

The use of telemedicine has grown significantly since the COVID-19 pandemic and has the potential to improve access to specialized care for otherwise underserved populations. Incarcerated people living with HIV (PLWH) could potentially benefit from expanded access to HIV care through telemedicine.

**Methods:**

All PLWH who were incarcerated within the Tennessee Department of Corrections and received care through the HIV telemedicine clinic at Regional One Hospital between 5/1/2019 through 2/28/2022 were identified from the electronic health records (EHR). Demographics, laboratory data, vaccine history, and treatment outcomes were abstracted from the EHR. Retention in care and viral suppression were defined using Centers for Disease Control and Prevention definitions.

**Results:**

Of the 283 incarcerated PLWH receiving care from this telemedicine clinic, 78% remained retained in care and 94% achieved or maintaining viral suppression at 12 months. Many preventative care measures remained unperformed or undocumented, including vaccinations and testing for concurrent sexually transmitted infections. There were 56 patients (20%) found to have chronic hepatitis C in this population, with 71% either cured or still on treatment in this study period.

**Conclusions:**

Retention in care and viral suppression rates were excellent among incarcerated PLWH receiving telemedicine care for their HIV. HIV related primary health care screenings and vaccinations, however, were less consistently documented and represent areas for improvement.

## Background

In the United States, especially the southern United States, incarceration and HIV represent dual burdens that often disproportionately affect already-underserved populations [1, 2]. While the prevalence of people living with HIV (PLWH) within the correctional system is higher than that of the general population, previous studies of PLWH within the correctional system have suggested the rate of disease progression decreases while incarcerated [3]. This improvement in outcomes, as compared to the general population, has been attributed to improved engagement along the HIV care continuum including improved HIV therapies and access to the health care system [4].

The HIV care continuum describes the progression through stages of treatment for individuals who have HIV, from diagnosis to prescription of antiretroviral therapy (ART), retention in care, and viral suppression [5, 6]. It is well known that PLWH engaged in the HIV care continuum have improved health outcomes. However, incomplete engagement in the HIV care continuum is common in the US representing the largest percent of HIV-infected individuals [6]. While engagement along the care continuum improves during incarceration, post-incarceration involvement drops, with participation sometimes less than pre-incarceration [7, 8]. Strategies for maintaining the engagement with care achieved during incarceration are needed.

With increased utilization of telehealth since the COVID-19 pandemic, specialized care, such as that for PLWH, is potentially more accessible for otherwise underserved populations. A recent study of specialized care delivery via telemedicine among underserved populations showed equivalent outcomes to in-person care with high patient satisfaction [9]. Other examinations of HIV-specific care delivery via telemedicine over the course of the COVID-19 pandemic showed improved visit completion, particularly among groups with lower pre-pandemic engagement during the height in 2019 [10]. However, continued study among the same cohort saw a return to predominantly in-person care delivery with persistent differences in telehealth utilization by subgroup [11].

In this study, we examine the care of PLWH who were incarcerated within the Tennessee Department of Corrections (TDOC) that were seen in the HIV telemedicine clinic at Regional One Hospital in Memphis, TN between 5/1/2019 through 2/28/2022. We describe the rates of adherence to HIV primary care and healthcare maintenance guidelines. This study also describes the prevalence and treatment outcomes of hepatitis C virus (HCV) infection within this population. This study aims to evaluate the delivery of specialized HIV care via telemedicine for this vulnerable population to better characterize the potential benefits and limitations of future telemedicine and correctional healthcare models.

## Methods

### Study population

This retrospective study included all PLWH incarcerated within the TDOC system that received care from Regional One Health from 5/1/2019 through 2/28/2022 for HIV via the telemedicine clinic. The Regional One Health system cares for a primarily underserved population in the Memphis metropolitan area in Tennessee and is a primary affiliate of the University of Tennessee Health Science Center. Through a contract agreement between Regional One Health and the Tennessee Department of Corrections, infectious disease specialists at Regional One Health provided remote HIV specialty care for all PLWH who are incarcerated within the TDOC system during the entire study period. There were no in-person clinic visits by our providers, only telemedicine visits throughout the study period. This is accomplished through HIPAA-compliant video software to allow for patient-provider interactions remotely. This software made it possible for the provider, who was located on campus at Regional One Health, to have audio and visual connection with the incarcerated patient and a nurse who both remained in the TDOC facility. The patient and nurse had audio and visual connections, being able to see and hear the provider. The nurse provided vital signs and assisted with the encounter as needed.

The data was accessed in May 2022 and all individuals receiving care with the TDOC as the encounter payor source were included for analysis. An individual patient could be released and reimprisoned multiple times but only counted as one individual patient in the data analysis to avoid duplicates. Patient demographics, laboratory data, treatment, and outcomes were abstracted from the EHR. This work was reviewed and approved by the University of Tennessee Health Science Center institutional review board and Regional One Health Office of Medical Research before any research activities were performed.

### Study variable and outcome definitions

The definitions for baseline demographics are as follows. Race and ethnicity were not consistently recorded so were not included. Gender, sexual practices, and substance use were obtained by self-report and abstracted from the EHR. Injection drug use (IDU) was defined as either current or in the past.

The primary outcomes for HIV care included retention in care and viral suppression, based on CDC definitions [5]. Retention in care was defined as attending at least two visits more than 90 days apart within the first year of follow-up. Continuous retention in care was defined as attending at least two visits more than 90 days apart for all years an individual received care within the study period. Viral suppression was defined as achieved if the patient had an HIV viral load of < 200 copies/mL at one year, defined as the closest visit 12 months after the first study visit, within a 3-month window. Continuous viral suppression was defined as an HIV viral load of < 200 copies/mL at all study visits after the initial visit.

Additional items related to the delivery of primary care for PLWH were chosen based on endorsed guidance [12] and are defined as follows. Lipids were defined as checked if lipid panel results were recorded at any point during the study period. The A1c was defined as checked within 12 months of the first visit. Tuberculosis screening was defined as being checked via tuberculin skin test or interferon gamma release assay at any point during the study period. Screening for syphilis with a rapid plasma reagin test (RPR) and for Gonorrhea/Chlamydia with urine nucleic acid amplification tests was marked as done if checked at any point during the study period. Vaccinations were defined as not documented or given if no documentation could confirm administration, documented as given in the past if records indicated administration prior to the study period, and given if doses were given during the study period.

Outcomes related to viral hepatitis are defined as follows. Hepatitis A virus (HAV) immunity was defined as a documented positive HAV antibody. Hepatitis B virus (HBV) immunity was defined as documentation showing a positive HBV surface antibody. HCV infection was defined as having a detectable HCV viral load by polymerase chain reaction (PCR). Treatment against HCV was done using direct-acting antiviral medications based on current guidelines [13]. Cure of HCV was defined as an undetectable HCV viral load by PCR after completion of targeted treatment. Re-infection of HCV was defined as a newly detectable HCV viral load by PCR after documentation of an undetectable viral load following treatment.

### Data analysis

All statistical tests were performed using SAS software, version 9.4 (SAS Institute, Cary, NC). A descriptive analysis of all participants was performed. Frequency analyses were then performed to assess the HIV care outcomes, primary care outcomes, and the viral hepatitis outcomes. The HIV care continuum, outlining the proportion of patients retained in care and virally suppressed at 12 months, and the HCV care continuum, outlining the proportion of patients with chronic HCV that were treated and cured, were then graphed.

## Results

A total of 283 PLWH were seen via telemedicine clinic from the TDOC, as seen in Table 1. Patients overwhelmingly identified as male (84%) with a mean age of 44 years. Risk factors relevant for HIV transmission were documented, including 96 inmates with a history of injection drug use and sexual practices for 155 inmates. There were 52.08% of participants reporting a history of IDU and 22.66% identified as men who have sex with men (MSM).

**Table 1:** Demographics of tennessee department of corrections inmates receiving telemedicine care for HIV (*N* = 283)

Characteristic	
Age, years, mean (SD)	44 (10.66)
Gender, N (%)	
Man Woman Transgender Woman	238 (84.10) 35 (12.37) 10 (3.53)
History of Non-Injection Illicit Drug Use, N (%), *N* = 25	17 (68)
History of Injection Drug Use, N (%), *N* = 96 Opiates, *N* = 19 Methamphetamines, *N* = 19 Cocaine, *N* = 19	50 (52.08) 5 (26.32) 10 (52.63) 4 (21.05)
Sexual Practices, N (%), *N* = 155	
None MSW WSM MSM WSW Bisexual, pansexual, or other	6 (4.69) 75 (58.59) 12 (9.38) 29 (22.66) 0 (0.00) 6 (4.69)
CD4 count (cells/µL), mean (SD), *N* = 274	686 (383.15)
Suppressed viral load at study entry, N (%), *N* = 267^a^ Viral load if detectable at study entry (copies/mL), median (IQR), *N* = 44	237 (88.76) 2608.5 (161.5, 24794.5)
On ART at any time, N (%)	270 (95.41)
On ART at study entry, N (%) NNRTI PI INSTI Other^b^	258 (91.17) 41 (15.89) 28 (10.85) 166 (64.34) 23 (8.91)
ART changed during study, N (%) ART changed to a regimen based on:	83 (29.33)
None (taken off ART at patient request) NNRTI PI INSTI Other^b^ TDF to TAF	1 (1.2) 9 (10.84) 8 (9.64) 60 (72.29) 5 (6.02) 30 (36.14)
Follow up time, days, median, (IQR)	560 (155, 832)

Abbreviations: SD, standard deviation; MSW, man who has sex with women; WSM, woman who has sex with men; MSM, man who has sex with men; WSW, woman who has sex with women; IQR, interquartile range; ART, antiretroviral therapy; NNRTI, non-nucleoside reverse transcriptase inhibitor; PI, protease inhibitor; INSTI, integrase strand transfer inhibitor; TDF, tenofovir disoproxil fumarate; TAF, tenofovir alafenamide

^a^ Suppressed viral load defined as < 200 copies/mL

^b^ Other ART regimens included a combination of NNRTI, PI, and INSTI medications

At study entry, defined as the first visit via telemedicine within our study window, the mean CD4 count was 686 cells/µl (standard deviation (SD) 383 cells/µl). At this initial encounter, 88% (*n* = 237) of patients had a suppressed viral load, defined as < 200 copies/mL. While 95% of patients reported a history of antiretroviral therapy at any time, fewer (91%) were actively on ART at time of study entry. The most common class of ART was integrase strand transfer inhibitors (61%). For 83 patients (29.3%), ART was changed during the study period, including 36 inmates who were transitioned from tenofovir disoproxil fumarate to tenofovir alafenamide.

Preventative care measures, including routine metabolic and infectious screenings were documented inconsistently as shown in Table 2. While most patients received lipid screenings (83%, *n* = 235), results for syphilis via RPR and Gonococcus/Chlamydia via co-testing were documented for only 34% and 21% of inmates respectively. Vaccination records from the correctional system as well as the Tennessee Immunization Information System revealed that a slight majority of inmates lacked any documentation of immunization with the pneumococcal conjugate (58%), pneumococcal polysaccharide (57%) or the meningococcal (55%) vaccines.

**Table 2:** Preventative care measures and HIV care outcomes, *N* = 283

Routine metabolic and infectious screenings, *N* (%)	
Lipids A1c Tuberculosis RPR GC/Ct	235 (83.04) 45 (15.90) 110 (39.43) 97 (34.28) 60 (21.20)
Pneumococcal conjugate vaccine, N (%)	
Not documented or given Given Documented as given in the past	166 (58.66) 67 (23.67) 50 (17.67)
Pneumococcal polysaccharide vaccine, N (%)	
Not documented or given Given Documented as given in the past	163 (57.60) 51 (18.02) 69 (24.38)
Meningococcal vaccine, N (%)	
Not documented or given Given Documented as given in the past	158 (55.83) 83 (29.33) 42 (14.84)
Tetanus vaccine, N (%)	
Not documented or given Given Documented as given in the past	216 (76.33) 21 (7.42) 46 (16.25)
COVID-19 vaccine, N (%)	
Not documented or given Given Documented as given in the past	146 (51.59) 130 (45.94) 7 (2.47)
Retained in care, N (%)^a^	
At 12 months, *N* = 280 Continuously, *N* = 278	219 (78.21) 190 (68.35)
Virally suppressed, N (%)^b^	
At 12 months, *N* = 217 Continuously, *N* = 243	204 (94.01) 210 (86.42)

Abbreviations: RPR, rapid plasma reagin; GC/Ct, Gonococcus and Chlamydia

^a^ Retention in care defined as at least two visits > 90 days apart within 12 months

^b^ Suppressed viral load defined as < 200 copies/mL

Review of hepatitis testing and management showed that just over half of inmates seen in this clinic were tested and found to be immune to both HAV and HBV, as shown in Table 3. 25% of study patients were vaccinated for HBV during the study period. HCV antibody testing was performed for 73% of inmates, with 81 (28%) of inmates documented to have positive HCV antibody titers. 56 inmates (19%) were diagnosed with chronic HCV, 49 received treatment and 22 were cured. 7 remained on treatment at the end of the study period.

**Table 3:** Hepatitides prevalence and Hepatitis C treatment outcomes (*N* = 283)

Hepatitis A, *N* (%)	
Tested, not immune Tested, immune Not tested or documented	42 (14.84) 156 (55.12) 85 (30.04)
Hepatitis B, N (%)	
Tested, not immune Tested, immune Not tested or documented Vaccinated if not immune or documented	65 (22.97) 163 (57.60) 55 (19.43) 71 (25.09)
Hepatitis C Antibody, N (%)	
Negative Positive Not tested or documented	126 (44.52) 81 (28.62) 76 (26.86)
Hepatitis C PCR, N (%)	
Negative Positive Not tested or documented	45 (15.90) 52 (18.37) 186 (65.72)
Hepatitis C treatment outcomes, N (%)	
Chronic Received treatment, *N* = 56 Still on treatment at time of data collection, *N* = 56 Cured, *N* = 56 Re-infected, *N* = 33	56 (19.79) 49 (87.50) 7 (12.50) 33 (58.93) 5 (15.15)

Abbreviations: PCR, polymerase chain reaction

**Fig. 1:**
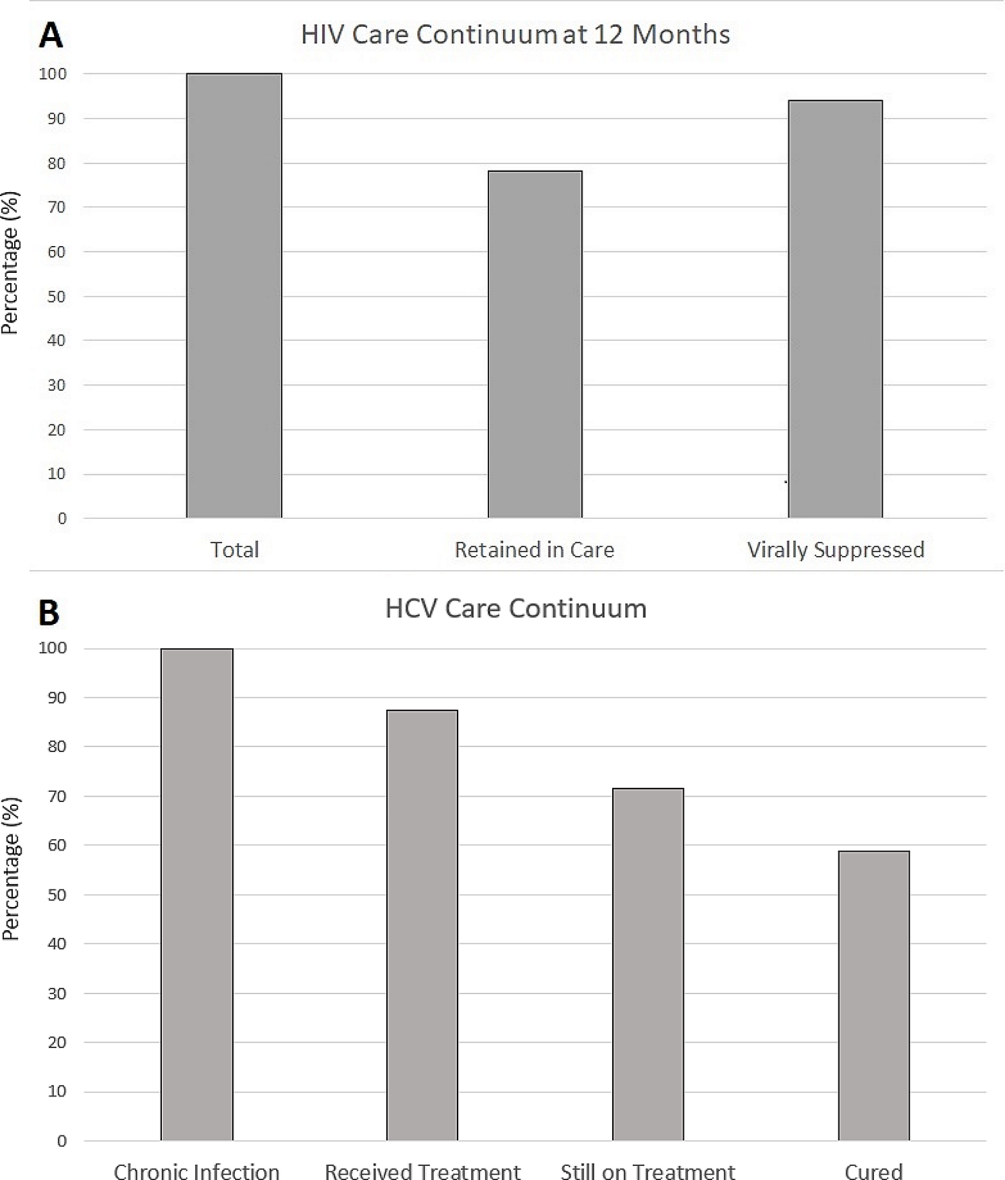
HIV and Hepatitis C care continua **Panel A**: proportion of patients (*N* = 283) retained in care (78%) and virally suppressed (94%) at 12 months. **Panel B**: proportion of patients (*N* = 56) who started hepatitis C treatment during the study period (88%), were still on treatment at the time of data collection (71%), and were cured (59%)

Longitudinally, this review found that 78% of patients were retained in care for at least 12 months, with 68% retained throughout the study period, as shown in Fig. 1. At 12 months, 204/217 (94%) of patients had achieved viral suppression, with 210 (86%) having continuous viral suppression throughout the study. The median amount of follow-up time per study participant was 560 days.

## Discussion

In this study evaluating 283 incarcerated PLWH receiving HIV care via telemedicine, we found excellent HIV care outcomes, with 78% retained in care and 94% being virally suppressed at 12 months. Of the 56 patients with Hepatitis C coinfection, 33 (59%) were cured during the study period with an additional 7 (13%) still on therapy at the time of data collection. We found these outcomes to be durable, with 210 (86%) having a continuously suppressed HIV viral load over a median follow-up time of 560 days. Our findings support the growing use of telemedicine in providing specialized care for patients, including incarcerated PLWH.

Being retained in care and achieving HIV viral suppression carries significant benefits both for the individual patient and for broader public health goals. Not only does HIV viral suppression provide numerous individual health benefits [14–17], including achieving a life-expectancy near that of people without HIV [18], it also lowers the risk of viral transmission through sex among heterosexual couples as well as MSM [19–21]. Additionally, previous work has seen that PLWH in the general population may often fall in and out of care, putting them at risk for poorer HIV care outcomes [22, 23]. We addressed this issue by looking at the standard definition of viral suppression at 12 months and continuous viral suppression throughout the study period and found consistently high rates of viral suppression.

While telemedicine use expanded significantly due to the COVID-19 pandemic, it is important to ensure equity is maintained. The Infectious Diseases Society of America and HIV Medicine Association put forth a policy paper calling attention to the need for more data evaluating the effects from implementing broader use of telemedicine services [24]. Several previous works have shown the use of telemedicine for PLWH to reduce barriers to care. One such work found that after transitioning to telemedicine visits, individuals who previously struggled to remain retained in care, including younger patients, Black patients, women, and those with detectable viremia, had improved retention in care when compared to pre-pandemic [10]. Telemedicine has also been shown to improve access to care for PLWH in traditionally underserved areas, particularly those living in rural or remote areas [25] and those with mental health or substance use disorders [26].

Unfortunately, other studies evaluating access to telemedicine visits have shown mixed results. Limited access to reliable telephone or video use may be contributing, as one study saw a drop in HIV outcomes, particularly among homeless individuals [27]. Another study, which showed positive effects of telemedicine among some populations, found that PLWH with limited access to reliable communication devices and those with lower technology literacy had poorer telemedicine uptake [26]. While telemedicine boasts the ability to have video meetings and provide insight into a patient’s living situation [28], in practice telephone calls rather than video appointments may predominate, with phone-only appointments accounting for 40-70% of telemedicine visits [10, 26]. Although there is some evidence that patients are equally happy with phone-only visits [29], other studies have found patients to prefer video or in-person visits to phone calls alone [30].

While most of the current literature focuses on the use of telemedicine to improve access to HIV specialized care to disadvantaged populations in the general population, there is currently sparse data evaluating the use of telemedicine for PLWH who are incarcerated. Most prior works are either proof-of-concept from the pre-COVID pandemic era [31] or have a shorter follow-up period [32]. Our work adds to the growing body of literature supporting the use of telemedicine to reach incarcerated PLWH by showing consistent HIV care outcomes over a median follow up of 560 days on a large scale. We also show the ability of the prison system to successfully identify, treat, and cure Hepatitis C infections with over two thirds of our patients being cured or still on treatment at the time of data collection.

Although we observed excellent HIV care outcomes in our cohort, many of the other primary care screenings and preventative healthcare measures were inconsistent. Current guidance recommends screening regularly for metabolic complications of HIV, concurrent sexually transmitted infections, and administering a variety of vaccines [12]. We observed infrequent testing of the majority of these screening tests and infrequent documentation of vaccinations. This may be related to a limitation of telemedicine, that the provider only has access to the medical records made available to him or her. Some of these tests may have been obtained and vaccinations given during our study period but not made available in the records shared with the providers. We tried to account for this by accessing the state vaccination database, but unfortunately this is an imperfect system and is not always up to date.

Another limitation of our work is that we only included inmates from Tennessee. While our center provided care for all PLWH in the TDOC system state-wide, this does limit applicability of our findings to states with different demographics. We also lacked key pieces of demographic information due to the limitations of the chart review, including race/ethnicity. Future studies evaluating the use of telemedicine in other states with different demographics would be helpful as telemedicine efforts continue to scale up. Additionally, we were not able to assess our patients’ opinions of their care by telemedicine. While other studies have evaluated patient attitudes toward telemedicine for HIV care, data is lacking for PLWH who are incarcerated. Further study evaluating patients’ satisfaction with their telemedicine care for incarcerated PLWH is also needed.

Among the many changes the COVID-19 pandemic brought to the medical community, the broader use of telemedicine for routine care has proven to be a beneficial development for many patients. Incarcerated PLWH have traditionally faced several barriers to care, but we found that telemedicine allowed for specialized care for this unique patient population. We were able to achieve excellent HIV care outcomes with viral suppression rates of over 90% at 12 months. Our work supports the use of telemedicine for the delivery of care to populations who would otherwise struggle to be seen by a specialist, including incarcerated PLWH.

## Data Availability

The datasets used and/or analyzed during the current study are available from the corresponding author on reasonable request.

## Abbreviations

PLWH: People living with HIV
HER: Electronic health records
ART: Antiretroviral therapy
TDOC: Tennessee Department of Corrections
HCV: Hepatitis C virus
IDU: Injection drug use
RPR: Rapid plasma reagin test
HAV: Hepatitis A virus
HBV: Hepatitis B virus
PCR: Polymerase chain reaction
MSM: Men who have sex with men
SD: Standard deviation
